# The Timecourse of Activation Within the Cortical Network Associated with Visual Imagery

**DOI:** 10.2174/1874440000701010001

**Published:** 2007-09-20

**Authors:** Sharlene D Newman, Donghoon Lee, L Christopher Bates

**Affiliations:** Department of Psychological and Brain Sciences, Indiana University, Bloomington, IN 47405, USA

## Abstract

The current study examined the hemodynamic timecourse of activation within a network of regions that is thought to be associated with visual imagery. Two experimental conditions were examined that were designed to place differential demands on specific nodes within the visual imagery network. The two tasks were an object inspection task and a mental rotation task. The two conditions recruited overlapping cortical regions; however several regions revealed a differential response to object inspection and mental rotation. The mental rotation condition elicited greater activation in parietal cortex, lateral occipital/temporal regions, and bilateral prefrontal cortex. Conversely, the object inspection condition elicited greater activation in inferior extrastriate cortex, the inferior frontal gyrus, and the right cerebellum. When examining the timecourse of activation three different timecourse patterns were observed across cortical regions and conditions. The shape of the hemodynamic timecourse appears to correspond strongly with the cognitive processing taking place within the region, not the stimulus paradigm. The paper discusses the significance of those varying timecourse shapes and has implications for the appropriateness of using the canonical hrf during fMRI data analysis.

## INTRODUCTION

Mental imagery is the ability to generate and manipulate mental representations, representations made available by memory. The study of mental imagery, in particular visual imagery, has a long and rich research history. There are many studies, including neuroimaging studies that have investigated this cognitive process. The current study was designed to take a closer look at the neural architecture that supports visual imagery by examining two processes that have been shown to require the use of visual imagery, visual object property inspection [1,2] and mental rotation [3-5]. The objective of this study was to examine the visual imagery network by observing how it responds under varying task demands. This will hopefully allow for better characterization of the processing taking place at each node.

Visual imagery, again, involves the generation of a mental representation that is no longer perceptually present. This means that this process involves the retrieval of object (or image) information from a memory store, generating and then holding that mental representation within a short-term buffer. When examining the neural network that supports visual imagery it is found that that network includes regions in very disparate locations within the brain, including medial occipital cortex, ventral temporal/occipital cortex, parietal cortex and prefrontal regions [4-6]. The function of each region is thought to be unique. The parietal cortex, particularly the region in and around the intraparietal sulcus (IPS), is thought to be responsible for visuo-spatial processing/working memory [7-11] while the inferior extrastriate cortex and the inferior frontal gyrus are thought to be associated with memory processes [12-15]. It has also been suggested that the parietal region collaborates closely with pre-frontal regions in the performance of working memory processes. In fact, it has been suggested that “parietal and frontal regions may mediate the retrieval of object representations from long-term memory, their maintenance in a working memory buffer, and the attention required to generate those mental images.” [6].

In one of the most famous studies of visual imagery Kosslyn [16,17] asked participants questions regarding the visual characteristics of objects (e.g., “Does a rabbit have a pink nose?”). The results of this study (participants respond faster when the rabbit is “bigger” in the image generated) have been taken as evidence to support the existence or use of visual imagery in cognition. Another important finding of this study and others like it is that the details of the image generated varies and that either a high or low resolution mental image can be created. In a meta-analysis attempting to distinguish between various models of visual imagery it was found that the involvement of early visual areas including the medial occipital cortex depended on whether or not the task required the imaged object to have high-resolution details [18]. Images with high resolution details may also be expected to depend more heavily on memory because more detailed information from memory must be retrieved. One of the experimental conditions in the current study was visual object property inspection. During this condition participants were required to generate a mental image of an object and then compare it to a second mental representation. For example, when presented with “The tip of a lizard’s tongue is shaped like the letter q.” participants must first generate a mental image of a lizard with its tongue out (a high resolution detail) and then a mental image of the letter Q to compare with the tongue. Therefore, while this object inspection condition is expected to activate the visual imagery network, it is also expected to heavily recruit medial occipital regions as well as the memory processing regions of the inferior ex-trastriate and the inferior frontal gyrus.

The second experimental condition examined was mental rotation. Mental rotation is a complex cognitive process that relies heavily on visual imagery. Conventional visual imagery involves the generation and maintenance of images – retrieving information from memory, generating an image, and holding it in a short-term buffer. Mental rotation, while also requiring the generation of a mental image, additionally involves the manipulation or transformation of that image. For example, when presented with “The capital letter h rotated 90 degrees is a capital i.e participants must generate a mental image of “H” and then rotate that image before comparing the transformed “H” to the generated image of “I”. Mental rotation has been found to involve a network of cortical regions that overlap significantly with visual imagery which includes parietal regions [4,5,8]; and visual processing regions, both early (see Kosslyn & Thompson, 2003 for a review) and higher visual processing regions [19]. Mental rotation has also been found to involve motor systems of the frontal cortex [3,20,21]. Because IPS is thought to be the visuo-spatial workspace, it is expected to be heavily involved during mental rotation. Therefore, while the object inspection task was expected to rely on medial occipital and inferior extrastriate cortex, mental rotation was expected to rely heavily on the parietal cortex (and frontal/motor processing regions).

Here, visual imagery will be elicited with the use of language stimuli. Previous studies have indicated that mental imagery generated from verbal instructions and from visual encoding activate similar cortical regions [22-25]. For example, a study conducted by Just and colleagues [26] compared the cortical activation when participants either read or listened to high- and low- imagery sentences. The high-imagery condition elicited greater activation in parietal and prefrontal regions similar to those observed in previous mental imagery studies [1,5,6,22,27,28]. In sum, language stimuli, in particular sentence stimuli, has been shown to elicit mental images in much the same way as non-verbal stimuli.

The goal of the current study was to better characterize the neural network involved in visual imagery by examining tasks that place varying demands on different nodes of the imagery network. In order to accomplish this goal we presented sentences that were designed to require either the inspection of high resolution images or the transformation of an image. Although great overlap of the processing modules (brain regions) was expected for these two conditions, the temporal dynamics of the response in these modules may vary as a function of task. Therefore, hemodynamic time-course analysis will be the primary analysis performed and is the primary focus of this study.

## METHODS

### Participants

The participants were 15 right-handed college students (7 females) aged between 19 and 41 years (mean=23.2±5.2). All were right-handed, native speakers of American English. Each participant gave signed informed consent approved by the Indiana University Institutional Review Board.

### Procedure

The experiment consisted of a practice session and a scanning session. In the practice session, participants were familiarized with the experiment and with the imaging environment. The experiment consisted of two conditions (object inspection and mental rotation) in which true/false statements were presented. For both the object inspection and mental rotation conditions the first noun phrase of the statements is a description of an object. However, for the object inspection condition more complex and detailed information was required. Another major difference between the two conditions is the verb phrase, or what participants are to do with the objects. In the object inspection condition (e.g., “The tip of a lizard’s tongue is shaped like the letter q.”) participants are expected to generate an image of the first object and compare it to the object described in the second noun phrase. On the other hand, in the mental rotation condition (e.g., “The capital letter h rotated 90 degrees is a capital i.”) participants are to manipulate (rotate) the first object and then compare it to the object described in the second noun phrase. The differences in processing occur at specific points in time which are expected to be observed in the fMRI time-course.

The stimuli used were piloted behaviorally. The pilot study consisted of a survey of 80 sentences. Participants were asked to state whether the sentences evoked a static visual image of an object (object inspection), a dynamic, or moving image of an object (mental rotation), or no image. They were then asked to rate how vivid the image generated was on a scale from 1-5 with 5 being extremely vivid. The sentences with a mean score of 3.5 or higher were used in the current imaging study; a total of forty sentences (20 for each condition) were selected. The mean number of words per sentence was similar for both conditions (object – 13.2; mental rotation – 13.5).

During scanning the sentences were presented on a screen located behind the participants and viewed *via* a mirror located on the head coil. Each sentence was presented for 8 seconds. Responses were collected using a fiber optic response device. The experiment was a slow, event-related design to ensure that the full hemodynamic response was accurately acquired. Each trial was separated by a 12-second rest period in which participants fixated on a plus sign in the center of the screen. The experiment consisted of two 8.75 min runs each run contained three 24-second fixation baseline periods.

### fMRI acquisition and analysis

The images were acquired on a 3T Siemen’s TRIO scanner with an 8-channel radio frequency coil located in the Imaging Research Facility at Indiana University. The functional images were acquired in 18 5mm thick oblique axial slices using the following parameters: TR=1000msec, TE=25 msec, flip angl=60^°^, voxel size=3.125mm x 3.125mm x 5mm with a 1mm gap.

The data were analyzed using statistical parametric mapping (SPM2 from the Wellcome Department of Cognitive Neurology, London). Images were corrected for slice acquisition timing, resampled to 2 x 2 x 2 mm voxels Images were subsequently smoothed in the spatial domain with a Gaus-sian filter of 8 mm at full-width at half maximum. The data were also high-pass filtered with 1/128 Hz cutoff frequency to remove low-frequency signals (e.g., linear drifts). The images were motion-corrected and the motion parameters were incorporated in the design estimation. The EPI data were normalized to the Montreal Neurological Institute (MNI) EPI template. At the individual level, statistical analysis was performed on each participant’s data by using the general linear model and Gaussian random field theory as implemented in SPM2. Each event (trial) was convolved with a canonical hemodynamic response function and entered as regressors in the model [29].

For the random effects analysis on group data, one-sample t-tests were performed on contrast images (task versus fixation, static versus dynamic, and dynamic versus static) obtained from each individual analysis. Activated brain areas surviving FWE (family-wise error) correction with a threshold of p<0.05 and a cluster extent threshold of 6 voxels (∼ were one voxel in the original image space) were rendered on a template brain in SPM2. The MNI coordinates associated with the peak activation within a cluster were transformed to Talairach coordinates. Talairach Daemon (http://ric.uthscsa.edu/projects/talairachdaemon.html) was used to obtain the location (Brodmann’s area) of each cluster.

Finally, timecourse analysis was performed. Regions of interest (ROIs) were defined as spheres (radius=10 mm) with the center at the peak coordinates of the activation clusters obtained from the group composite maps. The MarsBar Matlab toolbox [30, http://marsbar.courceforge.net] was used to extract ROI data from each individual. Timecourses were collapsed across trials for each task condition for each participant and baseline corrected. An analysis of variance (ANOVA) was applied at each point in the timecourse to determine condition effects. A Bonferoni correction was applied to account for multiple comparisons.

## RESULTS

**Behavioral**. The results indicated that the time to respond to the mental rotation condition (M=5673.08±595.3) was significantly longer than the object inspection condition (M=4705.8±513.6) [F(1,14)=69.31,p<0.001].

### fMRI Activation

**Conjunction Analysis**. A conjunction analysis was performed to determine the brain regions that were active during both conditions (see Fig. (**1)**). The regions that revealed significant overlap included: bilateral visual processing regions (both primary and secondary visual cortex) extending into the inferior temporal gyrus; a small region of the left BA7/40 of the parietal cortex; and a region of the left frontal cortex (BA6 extending inferiorly into BA44).

**Mental Rotation Minus Object Inspection**. A number of cortical regions were found to be more involved during mental rotation compared to object inspection. These regions included those that have been implicated in spatial processing – bilateral parietal regions (inferior parietal and the in-traparietal sulcal regions), bilateral occipital/temporal regions, and the right prefrontal cortex – as well as the left premotor cortex (see Fig. (**(2)** and Table **1**).

**Object Inspection Minus Mental Rotation**. Although the mental rotation task was found to be more difficult than the object inspection task, based on the increase in reaction time, there were a number of cortical regions found to be more involved during object inspection compared to mental rotation. These regions included those that have been implicated in semantic processing – the inferior frontal cortex (BA 47) and the left superior frontal cortex – as well as the right cerebellum and the medial occipital cortex (lingual gyri), bilaterally (see Fig. (**(2)** and Table **1**).

**Timecourse Analysis**. As expected, the two conditions activated a similar network of brain regions. However, the amount of activation within these regions was modulated by condition. This modulation can be seen easiest by examining the hemodynamic timecourse of activation. While there were a number of regions whose activation did not differentiate object inspection and mental rotation (e.g., early visual processing, regions of the inferior temporal cortex and a region of the right cerebellum, see Fig. (**3**)) there were others that did seem to activate more for one or the other condition. There were regions of the inferior temporal/occipital cortex, the medial superior extrastriate, the inferior frontal cortex, and the right cerebellum which revealed significantly more activation for the object compared to the mental rotation task (see Fig. (**4**)). Regions of the parietal cortex, inferior temporal/occipital cortex, and the prefrontal cortex revealed significantly more activation for the mental rotation compared to the object inspection condition (see Fig. (**5)**).

While these results mirror the activation cluster data, the timecourses provide additional information. As can be observed, not only are there amplitude differences between conditions, in some cases the shape of the hemodynamic response is also quite different across conditions and brain regions. In most of the timecourses, the onset of activation is 4-5 seconds after stimulus onset and the activation peaks between 12 and 14 seconds after stimulus onset. However, there are some regions that show a delayed onset of the hemodynamic response (e.g., the right prefrontal cortex – BA 10), and other regions that show two activation peaks, an additional earlier peak about 7 seconds after stimulus onset (e.g., medial superior extrastriate – BA 19). Finally, there were regions that revealed different shaped hemodynamic responses relative to the condition (e.g., right parietal cortex – superior BA 40). These differences in the timecourse across regions and conditions are discussed below.

## DISCUSSION

Our findings show that the object inspection task and the mental rotation task elicited differential effects on the neural network responsible for visual imagery. While both object inspection and mental rotation rely on a common cognitive-neuroanatomical network, the current findings indicate that object inspection relies more heavily on object memory processing associated with the inferior extrastriate and the inferior frontal gyrus. Conversely, mental rotation is more computational in nature and relies more on processes associated with the parietal and frontal cortices. Below we have attempted to relate the timecourse differences across regions and conditions to specific computations.

### Occipital/Temporal Cortex

There are a number of processing nodes within the visual cortex (e.g., early visual processing regions and regions within both the inferior and superior extrastriate) that appear to be involved in the current study. The involvement of early visual processing regions in the tasks used in the current experiment is expected due to the use of visual stimuli. No differential activation was predicted in these regions (BA17 and 18) and none was observed (see Fig. (**3**)). As shown, the timecourses for the object inspection and mental rotation conditions were very similar and lie almost exactly on top of each other.

In addition to early visual processing regions, several regions of the extrastriate cortex were found to be involved in the imagery network. Interestingly, three different time-course patterns were observed in extrastriate. In the regions that revealed a larger amplitude response for object inspection compared to mental rotation, the timecourse revealed two peaks (medial occipital regions including left and right lingual gyri and the medial superior extrastriate – see Fig. (**4A-C**). Both neuroimaging and lesion studies have implicated posterior ventral cortex, including inferior extrastriate (IES, which includes fusiform and lingual gyri), in object memory [12,13,32,33]. In fact, IES was found to be involved when subjects matched stimuli for semantic meaning as well as for structural properties suggesting that this occipital region is involved in a common memory network [32]. An explanation for the involvement of IES in object memory is that the region is involved in the processing of high-level perceptual descriptions of objects [13]. Again, in the current study the IES, specifically the bilateral lingual gyri, was found to be more involved during object imagery and it revealed the two-peak timecourse.

The early peak may be related to retrieving from memory information about the objects depicted in the question while the later peak may be related to evaluating the information retrieved and responding. Although both conditions elicited a response from the lingual gyri, the response was significantly greater for the object inspection compared to the mental rotation condition. This may reflect a more detailed, intensive search through memory for the object inspection condition. A similar pattern of activation was observed in this region in a previous study comparing haptic and visual imagery [34]. In that study greater reliance on the IES was observed during imagery of haptic object properties such as roughness and hardness compared to visual properties like shape and size. There the explanation for the difference in the involvement of IES was that making material judgments required the retrieval of more detailed object information from memory. A similar explanation can be given here in that the object inspection condition relies on the retrieval of more detailed object attributes than does mental rotation, which relies primarily only on shape information.

The more lateral regions of the occipital cortex, extending into the left middle temporal gyrus, unlike the medial occipital regions, showed a greater response to the mental rotation than the object inspection condition. The inferior occipito-temporal cortex revealed a hemodynamic time-course that peaked 14 seconds after stimulus onset (see Fig. (**5A-C**)). The region is similar to the location (similar Ta-lairach coordinates) found in previous object processing tasks focusing on shape, the lateral occipital complex [35-38]. One possible explanation for the current result is that although shape processing is a component of both object inspection and mental rotation, the mental rotation condition relies more on shape information. In addition, the manipulation component of the mental rotation task may be expected to place an additional demand on the shape orientation processing associated with the lateral occipital complex. There is some evidence to support this hypothesis. For example, in a recent mental rotation study using Cooper figures [39] of varying shape complexity, it was found that the activation in a similar region of the occipito-temporal cortex revealed not only main effects of shape complexity and amount of rotation, but also an interaction between the shape complexity and the amount of rotation [19]. These results indicate that shape processing is affected by mental rotation and suggests that the greater involvement of the region during the task in the current study may be due to the manipulation demands of the condition.

### Parietal Cortex

Several studies have shown that the region at the junction of the superior and inferior parietal cortex, the intraparietal sulcus (IPS), is involved in visuo-spatial processing, particularly when that image is manipulated. For example, the region has been routinely found to be activated during mental rotation tasks [4,5,19,24,27]. In the current study greater activation was observed in this region, bilaterally, for mental rotation compared to the object inspecition condition. The focus of that activation can be found in the superior portion of BA40, bilaterally. Interestingly, when examining the timecourses, these regions not only revealed a much larger amplitude response for mental rotation compared to object inspection, but the activation onset begins much earlier for mental rotation (see Fig. (**5D**,**5E**)). Again, this seems to indicate that the regions are involved in “determining the answer” (manipulation) for mental rotation, but only involved late (during evaluation and responding) for object inspection. While the parietal region has been found to be involved in mental rotation, it has also been found to be linked to image generation as well [6]. It may be that the early response for the spatial condition is associated with the generation and rotation of objects. For object inspection it may be that image generation occurs later, after the object properties have been extracted from memory. This hypothesis would account for the condition timecourse differences within the parietal cortex. However, further studies are necessary to disambiguate these two processes.

### Prefrontal Cortex

The prefrontal cortex, including bilateral BA 6 and right BA 10, was found to be more involved during mental rotation than object inspection. These regions have been long associated with spatial working memory processing [40–42]. For example in a study examining a group of stroke patients with lesions to the right middle frontal gyrus (which includes BA 10), it was found that these patients were impaired in their ability to keep spatial information on-line during the Corsi block-Tapping task [40]. In the current study onset of the hemodynamic timecourse in BA10 appears to occur late in the trial for both imagery conditions, during the evaluation and response portion of the trial (see Fig. (**5I**)). It may be that the region is involved in maintaining the visual images of the objects in memory.

In addition to BA 10, activation was also observed in BA 6 in and around the superior frontal sulcus, bilaterally. As mentioned above, these regions have been linked to spatial processing and have been observed during several visuo- spatial tasks. Portions of premotor cortex lies in BA6. As stated earlier, previous studies of the mental rotation task have found the involvement of motor processing regions [3,20,21]). In addition to mental rotation, another visuo-spatial task that has consistently recruited BA 6 is the Tower of London [TOL; 43,44]. In a TOL study in which goal hierarchy was manipulated while leaving the minimum number of moves constant, activation within bilateral BA 6 was observed [44]. This activation was thought to be associated with the spatial manipulation requirements of the task. Here, the involvement of BA 6 occurs early during the mental rotation trial, as indicated by the early onset of the hemodynamic timecourse (see Fig. (**5G**,**5H**)). In the case of the mental rotation task the early processing may be related to the imaged movement of the objects. This fits in well with its previously hypothesized role.

### Left Inferior Frontal Cortex

In addition to the involvement of the medial occipital regions, object inspection also elicited a greater response from prefrontal regions, particularly those that have been associated with semantic memory processing, the inferior frontal gyrus. Neuroimaging studies examining single word processing (e.g., verb generation tasks) have found that the inferior frontal gyrus, particularly BA47, is involved in semantic memory processing [14,15,45]. Petersen and colleagues [46], for example, examined a task in which subjects monitored a word list for instances of dangerous animals. Relative to a passive viewing baseline task, activation at or near BA 47 was found providing evidence for its role in semantic memory. In addition, during a verbal imagery task in which subjects listened to spatial words (e.g., “rotate”) and used them to assemble mental images, no inferior frontal gyral activation was detected [22]. This suggests that left BA47, as has been suggested previously [14], may be involved in controlled semantic retrieval processes; meaning the strategic retrieval and/or manipulation and evaluation of stored information. Furthermore, although both object inspection and mental rotation require the retrieval of information from memory, given that more detailed object attributes must be retrieved during object inspection, it may be expected that a greater degree of controlled retrieval processes is required for object imagery. The hemodynamic timecourse data also provides some evidence for this hypothesis. Left BA47 shows a much faster rise time for the object inspection compared to the mental rotation condition, suggesting that it is more involved early (when retrieving information) for object inspection and only later (when evaluating and responding) during mental rotation (see Fig. (**4D**)).

### Cerebellum

The right posterior lobe of the cerebellum, like the inferior frontal gyrus, revealed greater involvement for object inspection than mental rotation. The cerebellum has primarily been linked to motor processing. The timecourse observed in the cerebellum appears to support that associated function in that the activation onset is late for both conditions, when subjects would be making a motor response (see Fig. (**4F**)). However, several recent studies have suggested that the cerebellum is involved in a number of cognitive functions including object naming [47] and verbal working memory [48–50]. For example, in a study using transcranial magnetic stimulation (TMS) to disrupt the functioning of the right cerebellum in healthy adults, a decrease in the performance of a verbal working memory task was observed [50]. It has been suggested that the right cerebellum works together with the left inferior frontal gyrus during verbal working memory. However, further studies are required to determine the precise role of the cerebellum in imagery.

## CONCLUSIONS

The current study is unique in that it takes advantage of the dynamic recruitment of processing modules within a trial by examining the temporal dynamics of the hemodynamic response. Here these temporal dynamics were used to characterize the processing associated with the individual nodes within the visual imagery neurocognitive network. By doing so, the cognitive processing differences between object inspection and mental rotation become clearer as well as the functional roles of the cortical regions that make up the visual imagery network. To summarize the current findings, object inspection has been found to rely more on the controlled access to stored object knowledge and therefore more strongly recruits medial extrastriate regions and the inferior frontal gyrus. Conversely, mental rotation has been found to rely more heavily on spatial manipulation processes associated with the parietal and frontal cortex.

The timecourse data presented here provide support for a set of operating principles for cortical computation put forth by Newman and Just [51]. One of the principles is that the topology (cortical composition) of neurocognitive networks associated with a given task changes dynamically, adapting itself to the demands of that task. This suggests that the involvement of the processing nodes within a network may be expected to vary not only in their level of involvement (measured by activation amplitude), but also in the temporal dynamics of their involvement (determined by the shape of the hemodynamic timecourse). Here three distinct time-courses were observed across regions and conditions. These differences are thought to be due to fluctuations in computational demand. This is important because it suggests that examining the shape of the hemodynamic response may be as informative as examining its amplitude.

These timecourse results also bring into question the use of the canonical hrf in fMRI data analysis. Although the “canonically shaped” hrf was used in the SPM analysis to choose the activation clusters to examine, when the time-courses were extracted they did not all have a canonical shape. In a previous study examining the mental rotation task Ecker *et al*. (2006) observed this very problem stating that “none of the conventionally used fitting functions (e.g., gamma variate fitting function) provided an adequate fit to the observed HRFs” [3, pg. 442]. Because of this poor fit, examining the hemodynamic timecourse is important. This finding also implies that some regions that are actually involved during task performance may be missed because its shape does not adequately fit the canonical hrf shape.

## Figures and Tables

**Fig. (1):**
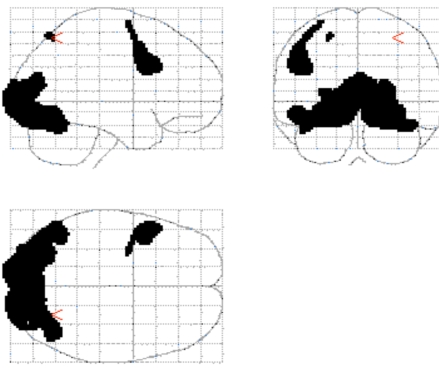
The conjunction map that shows the common activation for object inspection and mental rotation.

**Fig. (2):**
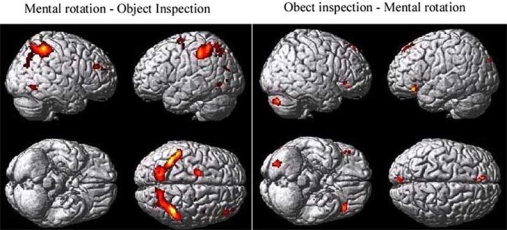
The group map showing the mental rotation minus object inspection contrast on the left and the object inspection minus mental rotation contrast on the right.

**Fig. (3):**
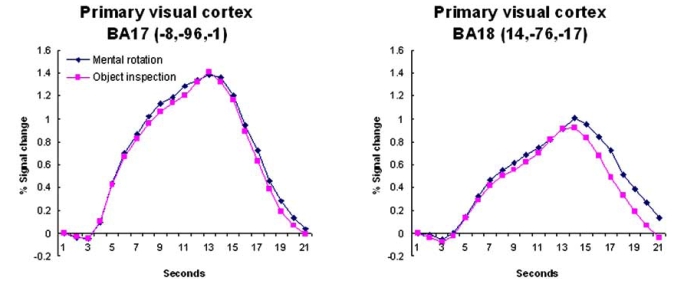
The timecourse data from regions that revealed no condition differences. These regions were located within early visual processing regions. The coordinates indicate the location of the cluster centroid.

**Fig. (4):**
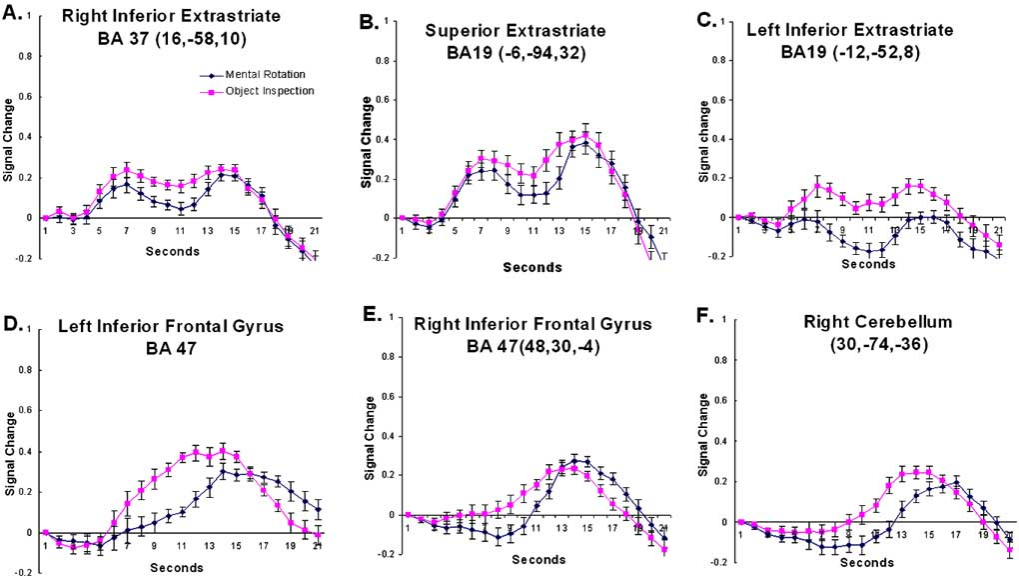
The timecourse data from regions that revealed a larger response to object inspection compared to mental rotation. The coordinates indicate the location of the cluster centroid. Error bars represent 95% confidence intervals based on the pooled Mse from the corresponding ANOVA [31].

**Fig. (5):**
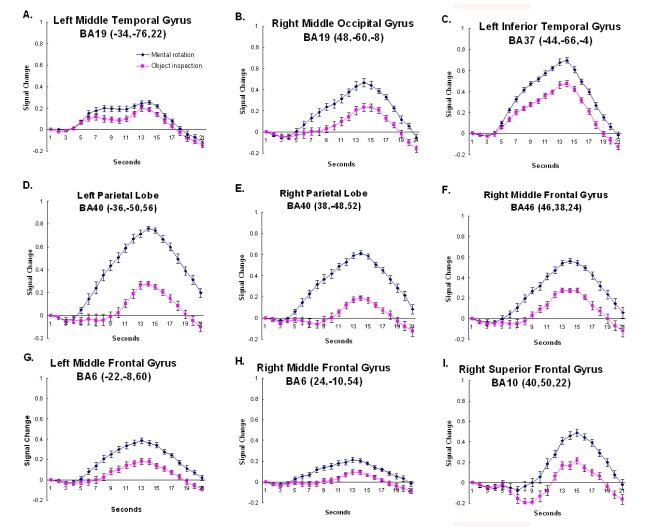
The timecourse data from regions that revealed a larger response to mental rotation compared to object inspection. The coordinates indicate the location of the cluster centroid. Error bars represent 95% confidence intervals based on the pooled Mse from the corresponding ANOVA [31].

**Table:** Table 1. Mental Rotation Minus Object Inspection

Cluster		Activation Peaks Within Cluster						
Region	Size	L/R	Anatomical Location	BA	MNI Coordinates			*Z* Value
					*x*	*y*	*z*	
Prefrontal cortex	67	R	Middle frontal gyrus	46	46	38	24	5.65
	231	L	Middle frontal gyrus	6	-22	-8	60	5.64
	18	R	Superior frontal gyrus	10	40	50	22	5.14
Parietal cortex	1857	L	Supramarginal gyrus	40	-36	–50	56	7.18
	1704	R	Supramarginal gyrus	40	38	–48	52	6.52
Temporal/Occipital Lobe	65	R	Middle occipital lobe	19/37	48	-60	-8	5.5
	29	L	Inferior temporal lobe	19	-44	-66	-4	5.17
	22	L	Occipito-temporal cortex	19	-34	-76	22	5.15

**Table:** Table 2. Object Inspection Minus Mental Rotation

Cluster		Activation Peaks Within Cluster						
Region	Size	L/R	Anatomical Location	BA	MNI Coordinates			*Z* Value
					*x*	*y*	*z*	
Prefrontal cortex	63	L	Inferior frontal gyrus	47	-44	28	-14	5.54
	54	L	Superior frontal gyrus	8	-8	42	52	5.39
	11	R	Inferior frontal gyrus	47	48	30	-4	5.38
Occipital Cortex	143	L	Lingual gyrus	30/19	-12	-52	8	5.64
	16	R	Lingual gyrus	30/19	16	-58	10	5.07
	45	L	Superior Extrastriate	19	-6	-94	32	5.34
Cerebellum	132	R	Posterior lobe		30	-74	-36	5.52

## References

[1] Kosslyn SM, Pascual-Leone A, Felician O (1999). The role of area 17 in visual imagery: convergent evidence from PET and rTMS. Science.

[2] Kline I, Paradis AL, Poline JB, Kosslyn SM, LeBihan D (2000). Transient activity inhuman calcarine cortex during visual imagery. J Cogn Neurosci.

[3] Ecker C, Brammer MJ, David AS, Williams SC (2006). Time-resolved fMRI of mental rotation revisited-dissociating visual perception from mental rotation in female subjects. NeuroImage.

[4] Carpenter PA, Just MA, Keller TA, Eddy WF, Thulborn KR (1999). Graded functional activation in the visuo-spatial system with the amount of task demand. J Cogn Neurosci.

[5] Just MA, Carpenter PA, Maguire M, Diwadkar V, McMains S (2001). Mental rotation of objects retrieved from memory: An fMRI study of spatial processing. J Exp Psychol Gen.

[6] Ishai A, Ungerleider LG, Haxby JV (2000). Distributed neural systems for the generation of visual images. Neuron.

[7] Newman SD, Just MA, Carpenter PA (2002). Synchronization of the human cortical working memory network. NeuroImage.

[8] Harris IM, Egan GF, Sonkkila C, Tochon-Danguy HJ, Paxinos G, Watson JD (2000). Selective right parietal lobe activation during mental rotation: a parametric PET study. Brain.

[9] D’Esposito M, Deter JA, Aguirre GK (1997). A functional MRI study of mental imagery generation. Neuropsychologia.

[10] Farah MJ, Levine DN, Calvanio R (1988). A case study of mental imagery deficit. Brain Cogn.

[11] Suchan B, Yaguez L, Wunderlich G (2002). Neural correlates of visuospatial imagery. BehavBrain Res.

[12] Kraut MA, Kremen S, Moo LR, Segal JB, Calhoun V, Hart J (2002). Object activation in semantic memory from visual multimodal feature input. J Cogn Neurosci.

[13] Humphreys GW (1996). Object recognition: The man who mistook his dog for a cat. Curr Biol.

[14] Wagner AD, Blagoev EJ, Clark J, Poldrack RA (2001). Left Prefrontal Cortex Guides Controlled Semantic Retrieval. Neuron.

[15] Gabrieli JDE, Desmond JE, Demb JB (1996). Functional magnetic resonance imaging of semantic memory processes in the frontal lobes. Psychol Sci.

[16] Kosslyn SM (1975). Information representation in visual images. Cogn Psychol.

[17] Kosslyn SM (1976). Can imagery be distinguished from other forms of internal representation? Evidence from studies of information retrieval time. Mem Cogn.

[18] Kosslyn SM, Thompson WL (2003). When is early visual cortex activated during visual mental imagery?. Psychol Bull.

[19] Koshino H, Carpenter PA, Keller TA, Just MA (2005). Interaction between the dorsal and ventral pathways in mental rotation: An fMRI study. Cogn Affect Behav Neurosci.

[20] Ganis G, Keenan JP, Kosslyn SM, Pascual-Leone A (2000). Transcranial magnetic stimulation of primary motor cortex affects mental rotation. Cereb Cortex.

[21] Vingerhoets G, de Lange FP, Vandemaele P, Deblaere K, Achten E (2002). Motor imagery in mental rotation: an fMRI study. NeuroImage.

[22] Mellet E, Tzourio N, Crivello F, Joliot M, Denis M, Mazoyer B (1996). Functional neuroanatomy of spatial mental imagery generated from verbal instructions. J Neurosci.

[23] Mellet E, Tzourio N, Denis M, Mazoyer B (1998). Cortical anatomy of mental imagery of concrete nouns based on their dictionary definition. Neuroreport.

[24] Mellet E, Bricogne S, Crivello F, Mazoyer B, Denis M, Tzourio-Mazoyer N (2002). Neural basis of mental scanning of a topographic representation built from a text. Cereb Cortex.

[25] Mazoyer B, Tzourio-Mazoyer N, Mazard A, Denis M, Mellet E (2002). Neural bases of image and language interactions. Intern J Psychol.

[26] Just MA, Newman SD, Keller TA, McEleney A, Carpenter PA (2004). Imagery in sentence comprehension: An fMRI study. NeuroImage.

[27] Mellet E, Tzourio-Mazoyer N, Bricogne S, Mazoyer B, Kosslyn SM, Denis M (2000). Functional anatomy of high-resolution visual mental imagery. J Cognitive Neurosci.

[28] Kosslyn SM, Shin LM, Thompson WL (1996). Neural effects of visualizing and perceiving aversive stimuli: a PET investigation. NeuroReport.

[29] Friston KJ, Holmes AP, Worsley KJ, Poline JP, Frith CD, Frackowiak RSJ (1995). Statistical parametric maps in functional imaging: a general linear approach. Hum Brain Mapp.

[30] Brett M, Anton JL, Valabregue R, Poline JB Region of interest analysis using an SPM toolbox [abstract]. Presented at the 8th International Conference on Functional Mapping of the Human Brain.

[31] Loftus GR, Mason MEJ (1994). Using confidence intervals in within-subject designs. Psychol Bull Rev.

[32] Vandenberghe R, Price C, Wise R, Josephs O, Frackowiak RSJ (1996). Functional anatomy of a common semantic system for words and pictures. Nature.

[33] Mutha S, Chertkow H, Beauregard M, Evans A (1999). The neural substrate of picture naming. J Cogn Neurosci.

[34] Newman SD, Klatzky RL, Lederman SJ, Just MA (2005). Imagining material versus geometric properties of objects: An fMRI study. Cogn Brain Res.

[35] Amedi A, Jacobson G, Hendler T, Malach R, Zohary E (2002). Convergence of Visual and Tactile Shape Processing in the Human Lateral Occipital Complex. Cereb Cortex.

[36] Amedi A, Malach R, Hendler T, Peled S, Zohary E (2001). Visuo-haptic object-related activation in the ventral visual pathway. Nat Neurosci.

[37] James TW, Humphrey K, Gati JS, Servos P, Menon RS, Goodale MA (2002). Haptic study of three-dimensional objects activates extrastria-te visual areas.

[38] James TW, James KH, Humphrey GK, Goodale MA, Heller MA, Ballesteros S (2005). In Touch and blindness. psychology and neuroscience.

[39] Cooper LA (1975). Mental rotation of random two-dimensional shapes. Cogn Psychol.

[40] van Asselen M, Kessels RCP, Neggers SFW, Kappelle LJ, Frijns CJM, Postma A (2006). Brain areas involved in spatial working memory. Neuropsychologia.

[41] Miotto EC, Bullock P, Polkey CE, Morris RG (1996). Spatial working memory and strategy formation in patients with frontal lobe excisions. Cortex.

[42] Owen AM, Downes JJ, Saahakian BJ, Polkey CE, Robbins TW (1990). Planning and spatial working memory following frontal lobe lesions in man. Neuropsychologia.

[43] Anderson JR, Albert MV, Fincham JM (2005). Tracing problem solving in real time: fMRI analysis of the subject-paced Tower of Hanoi. J Cogn Neurosci.

[44] Lee D, Newman SD, Just MA Timecourse of brain activity in the Tower of London Task. Presented at the Cognitive Neuroscience Society Conference.

[45] Fiez J.A (1997). Phonology, semantics, and the role of the left inferior prefrontal cortex. Hum Brain Mapp.

[46] Petersen SE, Fox PT, Posner MI, Mintun M, Raichle ME (1989). Positron emission tomographic studies of the processing of single words. J Cogn Neurosci.

[47] Price CJ, Moore CJ, Humphreys GW, Frackowiak RS, Friston KJ (1996). The Neural Regions Sustaining Object Recognition and Naming.Proceedings. Biol Sci.

[48] Desmond JE, Gabrieli JDE, Wagner AD (1997). Lobular patterns of cerebellar activation in verbal working memory and finger tapping tasks as revealed by functional MRI. J Neurosci.

[49] Desmond JE, Fiez JA (1998). Neuroimaging studies of the cerebellum: language, learning and memory. Trends Cogn Sci.

[50] Desmond JE, Chen SH, Shieh PB (2005). Cerebellar transcranial magnetic stimulation impairs verbal working memory. Ann Neurol.

[51] Newman SD, Just MA, Sternberg RJ, Pretz J (2005). In Cognition and intelligence: Identifying the mechanisms of the mind.

